# Peer Review of “Impact of COVID-19 Testing Strategies and Lockdowns on Disease Management Across Europe, South America, and the United States: Analysis Using Skew-Normal Distributions”

**DOI:** 10.2196/28681

**Published:** 2021-04-21

**Authors:** Gabriel Maia

**Affiliations:** 1 School of Sciences Osaka University Osaka Japan

**Keywords:** COVID-19, testing strategy, skew-normal distributions, lockdown, forecast, modeling, outbreak, infectious disease, prediction

*This is a peer-review report submitted for the paper "COVID-19 Testing Strategies and Lockdowns: The European Closed Curves, Analyzed by Skew-Normal Distributions, Forecasts for the United Kingdom, Sweden, and the United States, and the Ongoing Outbreak in Brazil."*

## Round 1 Review

### General Comments

This paper [1] is very interesting and approaches the problem at hand with an innovative perspective. The statistical analysis is quite enlightening, showing how the available data should be interpreted and used to improve health systems’ response to the crisis and explaining why the working strategies of countries like Germany and South Korea are so effective. I fully support the publication of this paper. I suggest only a more careful review to correct a few typos in the main text. Other than that, I endorse this paper’s publication.
